# Exploring brain functional connectivity in rest and sleep states: a fNIRS study

**DOI:** 10.1038/s41598-018-33439-2

**Published:** 2018-11-01

**Authors:** Thien Nguyen, Olajide Babawale, Tae Kim, Hang Joon Jo, Hanli Liu, Jae Gwan Kim

**Affiliations:** 1 Gwangju Institute of Science and Technology, Department of Biomedical Science and Engineering, 123 Cheomdan-gwagiro, Buk-gu, Gwangju 61005 Korea; 2 University of Texas at Arlington, Department of Bioengineering, 500 UTA Blvd, Arlington, Texas 76019 United States; 3 Mayo Clinic, College of Medicine, 200 First Street SW, Rochester, Minnesota 55905 United States; 4 Gwangju Institute of Science and Technology, School of Electrical Engineering and Computer Science, 123 Cheomdan-gwagiro, Buk-gu, Gwangju 61005 Korea

## Abstract

This study investigates the brain functional connectivity in the rest and sleep states. We collected EEG, EOG, and fNIRS signals simultaneously during rest and sleep phases. The rest phase was defined as a quiet wake-eyes open (w_o) state, while the sleep phase was separated into three states; quiet wake-eyes closed (w_c), non-rapid eye movement sleep stage 1 (N1), and non-rapid eye movement sleep stage 2 (N2) using the EEG and EOG signals. The fNIRS signals were used to calculate the cerebral hemodynamic responses (oxy-, deoxy-, and total hemoglobin). We grouped 133 fNIRS channels into five brain regions (frontal, motor, temporal, somatosensory, and visual areas). These five regions were then used to form fifteen brain networks. A network connectivity was computed by calculating the Pearson correlation coefficients of the hemodynamic responses between fNIRS channels belonging to the network. The fifteen networks were compared across the states using the connection ratio and connection strength calculated from the normalized correlation coefficients. Across all fifteen networks and three hemoglobin types, the connection ratio was high in the w_c and N1 states and low in the w_o and N2 states. In addition, the connection strength was similar between the w_c and N1 states and lower in the w_o and N2 states. Based on our experimental results, we believe that fNIRS has a high potential to be a main tool to study the brain connectivity in the rest and sleep states.

## Introduction

Functional connectivity measures the temporal correlations between anatomically separated brain regions. One of the pioneers in the study of the brain functional connectivity on humans was Biswal, who conducted an experiment during a resting state using functional magnetic resonance imaging (fMRI) in 1995^1^. Since then, fMRI has played an important role in the connectivity research in both healthy people and patients. Using fMRI, researchers have consistently reported several important networks in healthy subjects, including sensorimotor, visual, auditory, attention, and default mode networks^1–5^. In patients, these networks, especially the default mode network (DMN), have been found to be affected in some brain disorders and diseases. A decrease in the connectivity and a deactivation in the DMN were reported in Alzheimer’s disease^6,7^. Furthermore, an alteration of the DMN was revealed in depression^8^, dementia^9^, and schizophrenia^10^. In addition to the DMN, the temporal and sensorimotor networks were noted to be changed in dementia with Lewy bodies^11^ and multiple sclerosis^12^.

With more than twenty years of history and many important findings, fMRI has demonstrated its key role in the examination of the brain functional connectivity. However, fMRI is very expensive, it has a low temporal resolution, and it restricts patients who have metal implanted devices. An alternative modality to study the brain networking is functional near-infrared spectroscopy (fNIRS). Compared to fMRI, fNIRS has a relatively low cost, a high temporal resolution, and no subject restriction. In addition, the fNIRS system is usually portable, which enables bedside monitoring^13^. Furthermore, in terms of the hemodynamic responses, while fMRI provides only information about blood oxygenation level dependent (BOLD) change, fNIRS provides more information including oxy-hemoglobin (HbO), deoxy-hemoglobin (Hb), and total hemoglobin (THb)^14^.

Utilization of fNIRS to study the brain functional connectivity was initiated by White *et al*. in 2009^15^. In their paper, they used diffuse optical tomography to collect resting brain signals in the motor and visual cortices. They identified the motor and visual functional networks. After White’s work, many other groups have utilized fNIRS to investigate the resting-state functional connectivity in various brain regions and subjects. Similar to the results found using fMRI, the studies using fNIRS have revealed the networks of sensorimotor, visual, auditory, and language systems in the resting brain in the normal healthy subjects^15–19^. In addition to the resting-state, fNIRS has been used to investigate the brain functional connectivity during a vigilance task and in sleep deprivation subjects^20,21^. Furthermore, this technique was not only applied to the healthy participants, but Metzger *et al*. also proved the usefulness of using fNIRS to understand the brain networks in patients by their connectivity study with Alzheimer patients^22^.

Apart from the resting-state connectivity, the sleep connectivity also plays an important role in understanding the brain functional connectivity. However, so far, there has been little work using either EEG, fMRI, or both to investigate the cerebral connectivity during sleep, and the results found from these papers are still controversial. While most studies demonstrated a decrease, decoupling, or breakdown of the DMN during sleep^23,24^, Larson-Prior *et al*. established the preservation of this network throughout all the examined arousal states^25^. Hence, further investigation of the sleep connectivity is needed. In this study, we collect fNIRS signals and examine the brain connectivity during the rest and sleep phases. According to our best knowledge, this is the first report on the cerebral functional connectivity in the sleep phase using fNIRS. We believe that the present work will help to interpret the spontaneous activity of the brain in the rest and sleep states.

## Results

### Subjects’ states

The subjects underwent two rest and two sleep states; quiet wake-eyes open (w_o), quiet wake-eyes closed (w_c), non-rapid eye movement (NREM) sleep stage 1 (N1), and NREM sleep stage 2 (N2). Table 1 shows the time (in minutes) each subject experienced each state. All 18 subjects had a w_o state, 17 subjects had a w_c state, 12 subjects had an N1 state, and 7 subjects had an N2 state. Because the measurement time in the sleep phase was only 10 minutes, no subject experienced NREM sleep stage 3 (N3) nor REM sleep. Except for the w_o state, the time in other states varied among subjects.

**Table 1:** The duration of each state from 18 subjects (unit: minute).

Subject	1	2	3	4	5	6	7	8	9	10	11	12	13	14	15	16	17	18
w-o	5	5	5	5	5	5	5	5	5	5	5	5	5	5	5	5	5	5
w-c	10	10	8.5	10	5	10	5.5	1.5	1	9.5	2	5.5	0	3	0.5	10	10	0.5
N1	0	0	1.5	0	5	0	4.5	4.5	4	0.5	3	4.5	3	1.5	2.5	0	0	1.5
N2	0	0	0	0	0	0	0	4	5	0	5	0	7	5.5	7	0	0	8

### Whole brain connectivity

**Figure 1:**
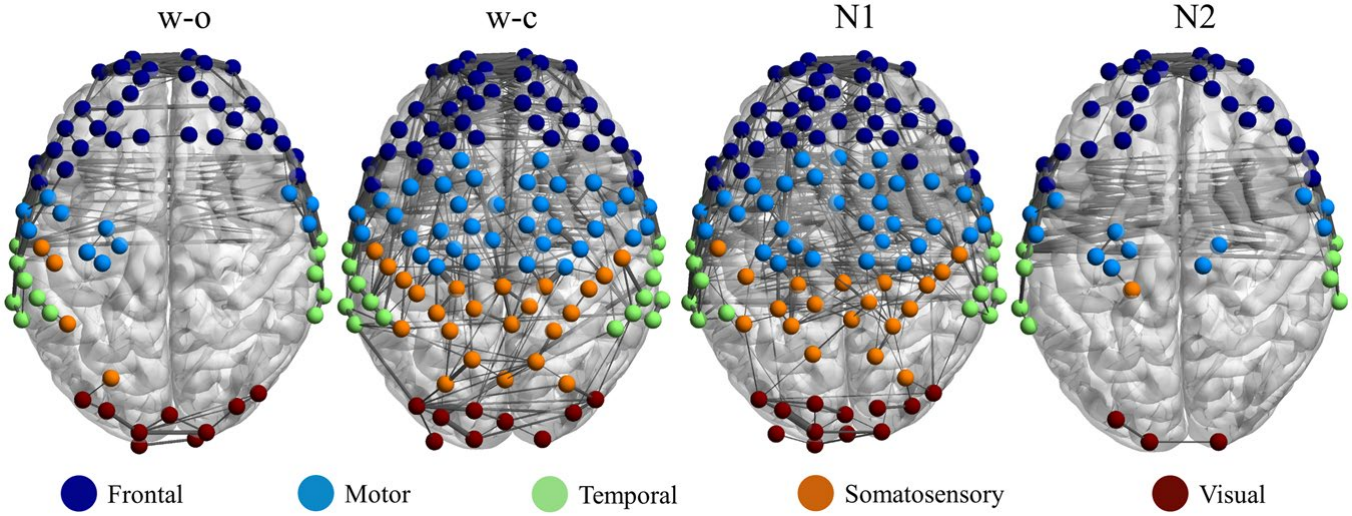
The group connectivity calculated from HbO in four states. Only connections with absolute z-values greater than 0.3 were displayed. Brain map images were generated using BrainNet Viewer software^38^.

In order to have a general view of the change of the connectivity in different states, we averaged the connectivity of the whole brain over all subjects. Though the threshold range was from 0.2 to 0.7, in Fig. 1, we displayed the brain map (calculated from HbO) with a threshold of 0.3 for the purpose of better visualizing the difference among states. After thresholding, the number of significant connections was high in the w-c and N1 states and lower in the w-o and N2 states in all networks. In addition, the somatosensory and its related networks altered most among the four states.

### Connection ratio (CR)

#### Oxy-hemoglobin (HbO)

**Figure 2:**
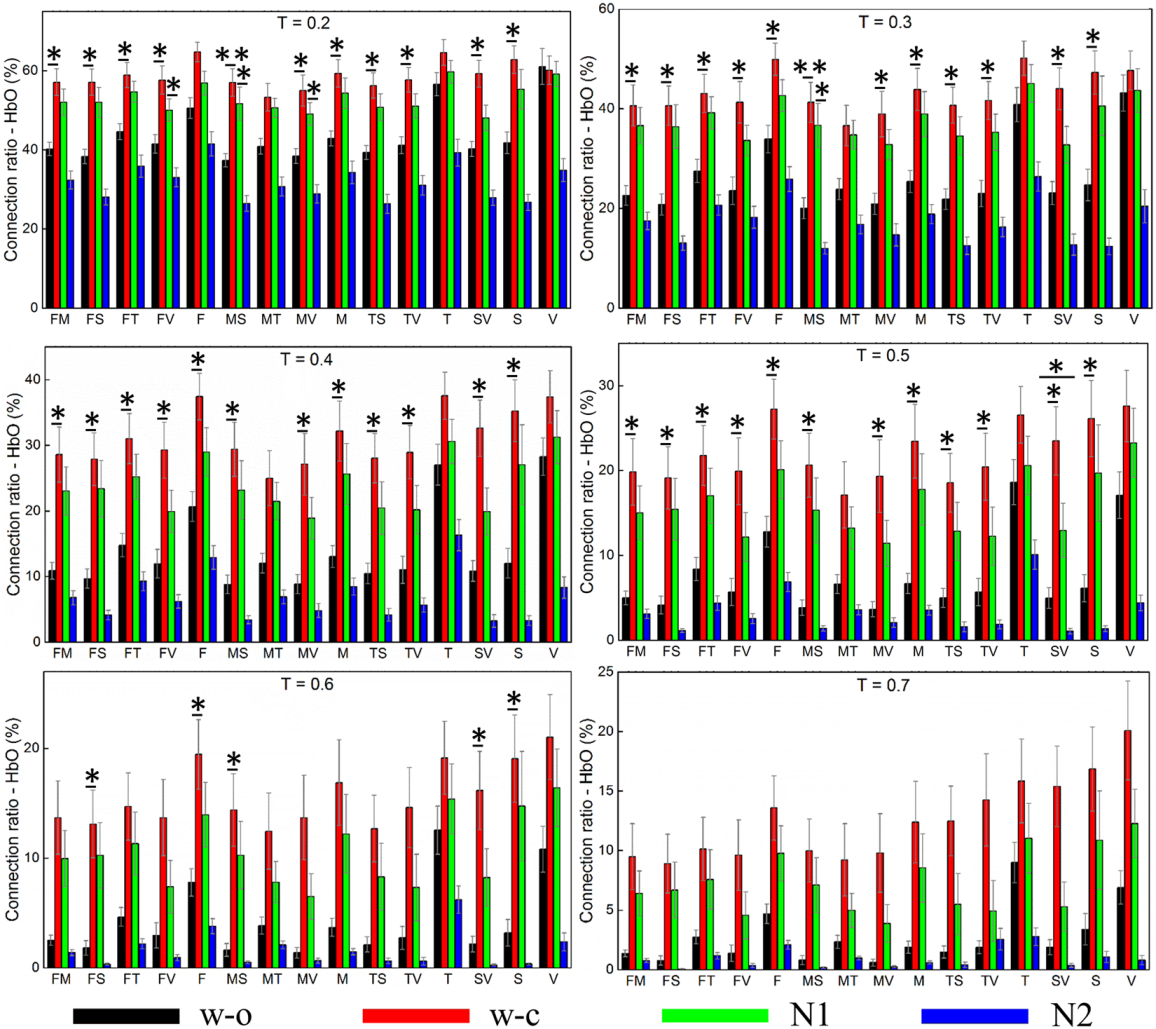
Connection ratios calculated from HbO for six different thresholds. The error bar represents the standard error. The marks indicate results from the statistical tests, *for statistical significance and **for highly statistical significance. T (T = 0.2): threshold; F: frontal, M: motor, T: temporal, S: somatosensory, V: visual, FM: frontal-motor.

Figure 2 displays the averaged connection ratio (CR) from 18 subjects derived from HbO for 15 networks in four states. In all 15 networks, the CR was high in the w_c and N1 states and low in the w_o and N2 states. When the threshold was low (from 0.2 to 0.5), the ANOVA test and the post-hoc *t*-test were statistically significant in most networks (except the visual, temporal, and motor-temporal networks). However, with a high threshold, the number of the networks that had a significant difference in different states decreased. At the threshold of 0.6, the statistical tests revealed a significant difference in the frontal, somatosensory, and their related networks. Additionally, the threshold of 0.7 resulted in no statistically significant difference among the four states.

#### Deoxy-hemoglobin (Hb)

**Figure 3:**
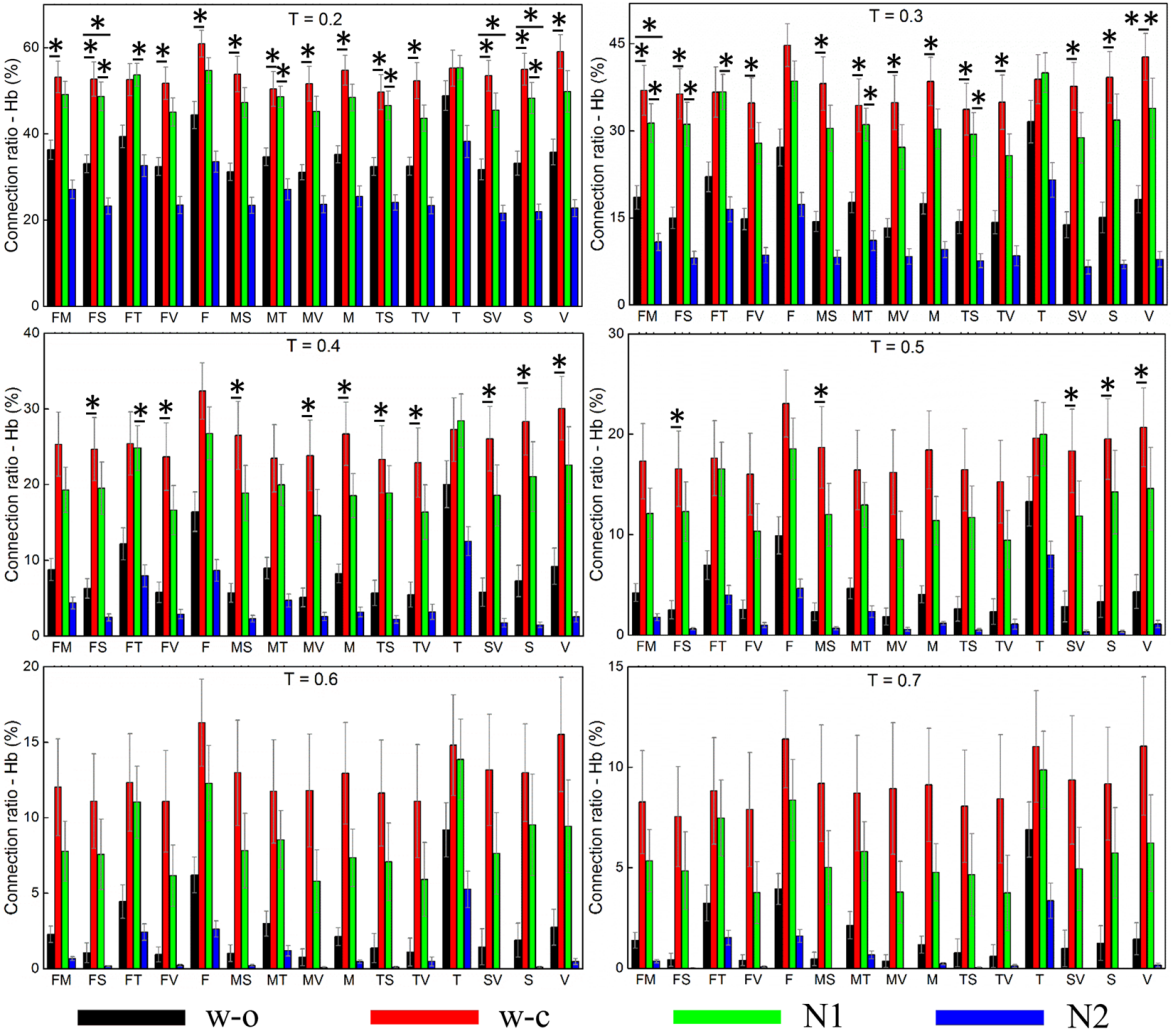
Connection ratios calculated from Hb for six different thresholds. The error bar represents the standard error. The marks indicate results from the statistical tests, *for statistical significance and **for highly statistical significance. T (T = 0.2): threshold, F: frontal, M: motor, T: temporal, S: somatosensory, V: visual, FM: frontal-motor.

Similar to the CR derived from HbO, the CR derived from Hb was high in the w_c and N1 states and low in the w_o and N2 states for all networks with all thresholds (Fig. 3). When the threshold was 0.2, the statistical tests were significant in all networks. However, the higher the threshold was, the less the number of the networks, which were significantly different among states, was. At 0.5 threshold, the significant difference was found in the somatosensory, visual, and their related networks. The threshold of 0.6 and 0.7 revealed no significant difference in any networks.

#### Total hemoglobin (THb)

In agreement with the other two hemodynamic responses, the CR derived from THb was highest in the w_c and N1 states, lower in the w_o state, and lowest in the N2 state with all threshold values. The ANOVA test revealed no statistical significance in any networks. A figure plotting the averaged CR of 18 subjects in 15 networks derived from THb is added as the Supplementary Fig. 1.

### Connection strength

#### Oxy-hemoglobin (HbO)

**Figure 4:**
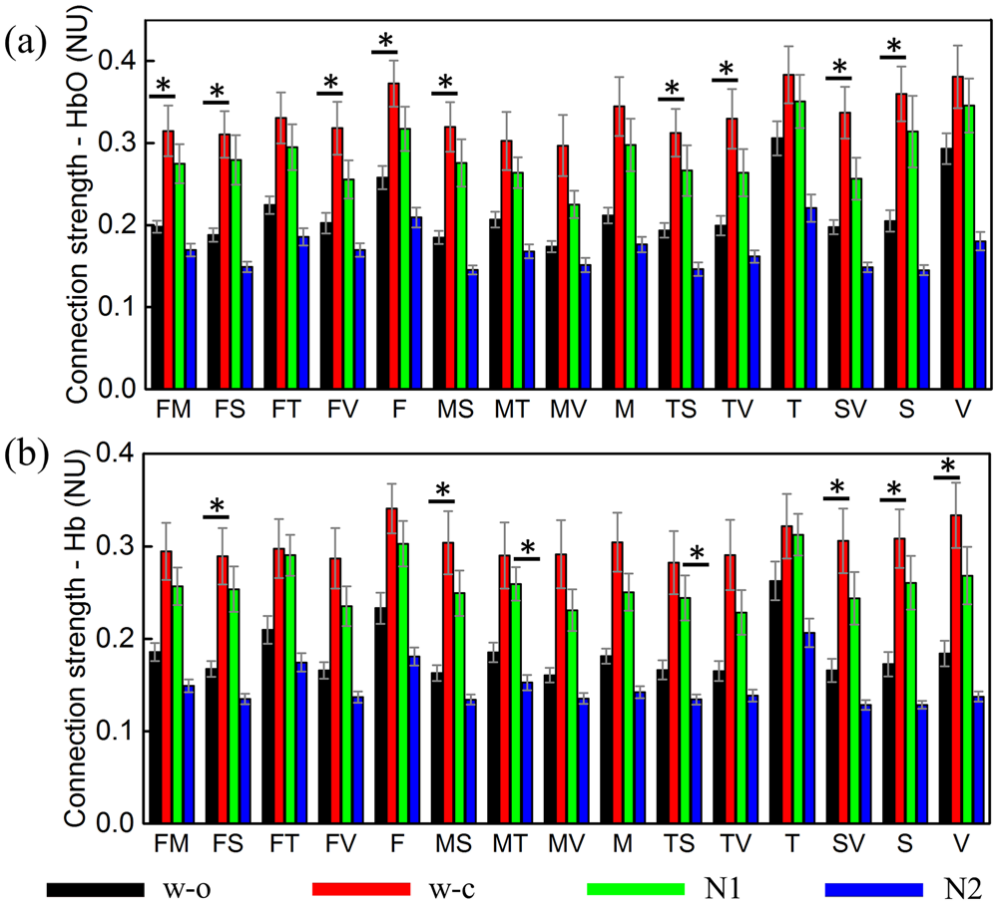
The averaged connection strength from 18 subjects in 4 states and 15 networks; (**a**) derived from HbO; (**b**) derived from Hb. The error bar represents the standard error. The marks indicate results from the statistical tests, *for statistical significance. F: frontal, M: motor, T: temporal, S: somatosensory, V: visual, FM: frontal-motor.

In all networks, the connection strength (CS) derived from HbO was similar between the w_c and N1 states, lower in the w_o state, and lowest in the N2 state (Fig. 4a). The ANOVA tests were statistically significant in the frontal, somatosensory, and their related networks. The post-hoc *t*-test revealed a significantly higher CS in the w_c state compared to the w_o states in these networks.

#### Deoxy-hemoglobin (Hb)

In agreement with the CR, the CS derived from Hb was high in the w_c and N1 states and low in the other two states for all networks (Fig. 4b). The ANOVA tests were statistically significant in the somatosensory, visual, somatosensory-related, and motor-temporal networks. The post-hoc *t*-test revealed a significantly higher CS in the w_c state than in the w_o state in the somatosensory, visual, and somatosensory-related networks. In addition, the CS in the N1 state was statistically significantly stronger than the CS in the N2 state in the motor-temporal and temporal-somatosensory networks.

#### Total hemoglobin (THb)

Unlike the CS derived from HbO and Hb, the averaged CS derived from THb was similar in the four states in all networks. The ANOVA tests revealed no statistically significant difference in any networks. The averaged CS of 18 subjects in 15 networks derived from THb is added as the Supplementary Fig. 2.

## Discussion

The brain functional networks were investigated in the rest and sleep states: w_o, w_c, N1, and N2. Fifteen networks formed from five brain regions were compared among four states using an ANOVA test and between two states using a post-hoc *t*-test based on the CR and CS. The brain functional connectivity was found to be strong in the w_c and N1 states and weaker in the w_o and N2 states. The statistical analysis revealed the statistically and significantly higher CR and CS in the w_c and N1 states compared to the w_o and N2 states in the somatosensory and its related networks.

The previous studies using EEG and fMRI reported an increase in the brain functional connectivity in light sleep and a decrease in these networks in slow wave sleep^23–27^. Larson-Prior found an increase in the functional connectivity in the dorsal attention network in light sleep^25^, and Horovitz *et al*. and Sämann *et al*. reported a breakdown of connectivity in the prefrontal cortex in slow-wave sleep^23,24^. Similar to the previous reports, our study showed that across all fifteen networks, the averaged CR and CS of 18 subjects were high in the w_c and N1 states and low in the N2 state. In detail, compared to the N2 state, the N1 state showed a significantly higher CS in the motor-temporal and temporal-somatosensory networks. Our findings of the decrease of the brain functional connectivity in the N2 state compared to the w-c and N1 states help to explain the reduced response of a sleep subject to the external environment.

In a comparison between the two resting states, we found the greater CR and CS in the w_c state than in the w_o state. In particular, a statistically higher CR was expressed in most networks, and a significantly stronger CS was shown in the somatosensory and its related networks in the w_c state. This finding was in agreement with the results reported by Brodoehl *et al*.^28^. Using fMRI, Brodoehl *et al*. found the enhancement of perception during eye closure.

We found that with high threshold, HbO was more sensitive to the connectivity difference across the states than Hb and THb. In fact, when the threshold was 0.6, the significantly higher CR was only found in the connectivity derived from HbO but not in the other two hemodynamic responses. In addition, the number of networks that expressed a statistical difference was highest in the connectivity derived from HbO. This difference may be due to the physiological differences of three hemodynamic response types^29^. The Wolf *et al*. study found a considerably lower connectivity in Hb compared to HbO. They explained it was because HbO was less influenced by the oxygen consumption. In addition, Wolf *et al*. stated that HbO was normally the most reliable indicator for the brain functional activity^29^. Furthermore, Strangman *et al*. through a simultaneous experiment of both fMRI and fNIRS reported the strongest correlation between fMRI changes and HbO^30^. They explained that the high correlation between HbO and fMRI was due to the superior contrast-to-noise ratio for HbO relative to Hb. As a result, though fNIRS cannot detect deep brain signals, its capacity to measure both HbO and Hb elevates its advantages over fMRI, which can assess Hb only^14^.

Our findings of the significant difference of the brain networks in the rest and sleep states suggest the potential of using fNIRS connectivity to score the sleep stages and further to verify anesthesia levels. In addition, our experimental results prove the ability of the fNIRS system in investigating the cerebral functional connectivity in the rest and sleep states. Although we measured fNIRS signals in the normal healthy subjects in this study, we believe that fNIRS technique can be applied to diagnose sleep disorder diseases by monitoring the patients’ brain connectivity during sleep as well as to predict the cognitive decline in dementia patients. The disadvantage of our study is that due to short recording time in the sleep phase, we were not able to collect data from NREM sleep stage 3 and REM sleep. Hence, we could not investigate the brain functional connectivity in these sleep stages. For future study, we recommend collecting sleep signals for a longer time, such as a whole night, if possible.

Physiological signals including EEG, EOG, and cerebral hemodynamic responses were measured simultaneously during the rest and sleep phases. The EEG and EOG signals were used to verify the subjects’ sleep stages, and the hemodynamic responses from fNIRS were utilized to compare the brain functional connectivity in terms of the connection ratio and connection strength among states. Analysis based on the connection ratio showed a significantly higher value in the wake-eyes closed state compared with the wake-eyes open state and the sleep stage 2 in the somatosensory and its related networks. On the other hand, a statistical test on the connection strength resulted in a significantly stronger connection in the wake-eyes closed state compared with the wake-eyes open state and the sleep stage 2 in the frontal, somatosensory, and their related networks. The similarity between our results from fNIRS and the previous results from fMRI suggests the potential of using fNIRS to investigate the brain functional connectivity in both rest and sleep with a low cost and high portability.

## Materials and Methods

### Participants, experimental setup and protocol

The experimental protocol was approved by the Institutional Review Board at the University of Texas at Arlington. All tests were performed in accordance with the relevant guidelines and regulations. A total of 18 subjects with the ages ranging from 18 to 28 (mean age 24), 15 males and 3 females, volunteered to participate in the experiment. Before the experiment, the experimental procedure was explained to each subject, and he/she signed an informed consent agreement. In addition, an informed consent for publication of identifying information/images in an online open-access system was obtained. Each subject also completed a questionnaire about his/her condition. All subjects declared that they had no major medical concerns and they had a sufficient amount of sleep (at least 7 hours) the previous night.

**Figure 5:**
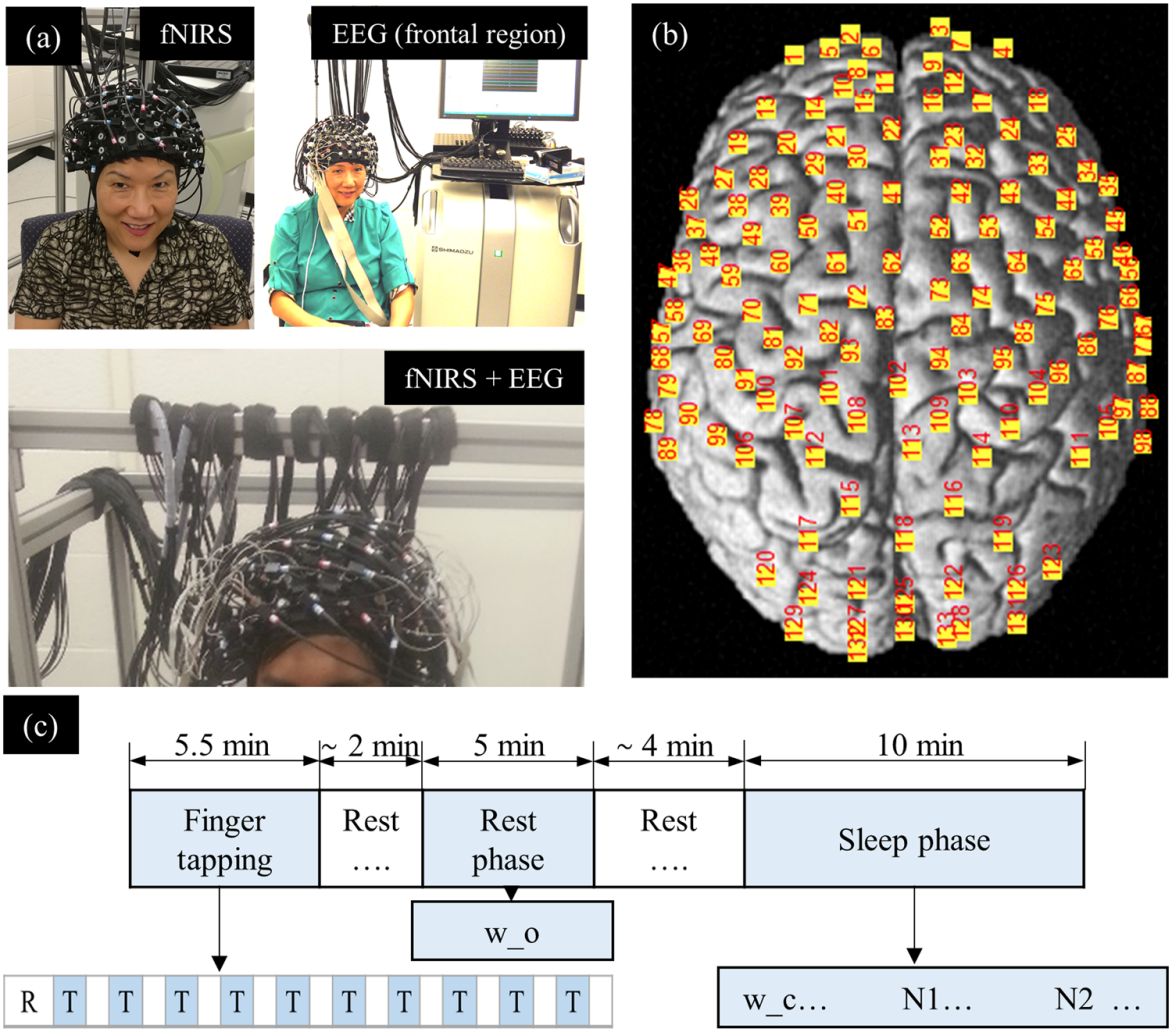
(**a**) Experimental setup; (**b**) The location of 133 fNIRS channels on the brain; (**c**) Experimental protocol.

The experiment was conducted every day at 1:30 p.m. over 18 days, one day per subject. During the experiment, the subject was seated in a comfortable chair with his/her neck laid on a pillow. In front of the chair, there was a table with a screen on it. The screen was half a meter away from the subject. The experimental protocol consisted of three phases: finger tapping, rest, and sleep phases (Fig. 5c). During the finger tapping phase, the subject was instructed to sit up straight, place his/her right hand on the table, and look at the screen to follow further instructions. This phase included the first 30-second resting and 10 consecutive finger tapping trials; each trial consisted of 20-second tapping and 10-second resting (Fig. 5c). When tapping, the subject used his/her right index finger. After the finger tapping, the subject was resting (approximately 2 minutes) while waiting for the operator to conduct the system checkup. Subsequently, the rest phase was performed. During the phase, the subject relaxed, opened eyes, and looked at a black cross at the center of a white screen. The rest phase lasted for 5 minutes (Fig. 5c). Both the finger tapping and the rest phases were conducted in a bright room, with all the lights turned on. After the rest phase, the subject was asked to relax, close his/her eyes, and try to sleep. All the lights in the room, including the screen, were turned off. We started to record signals in the sleep phase; the signs of sleep were often observed (prominent of the theta band in the EEG signals). The gap time between the rest and the sleep phase varied among subjects, most within 4 minutes (Fig. 5c). The sleep phase lasted for ten minutes (Fig. 5c). The averaged experimental time, including the setup time, was between two and three hours.

### Data recording

#### Hemodynamic response measurement

The hemodynamic responses were measured using the LABNIRS system (Shimadzu, Japan) with a sampling rate of 8.13 Hz. The fNIRS system consisted of 40 light sources and 40 light detectors. A source emitted the light at three wavelengths, 780 nm, 805 nm, and 830 nm. The sources and detectors were designed to form 133 channels, covering the whole head (Fig. 5a,b). The distance between a pair of a source and a detector was 3 cm.

#### Electrophysiological signal recording

The electroencephalography (EEG) and electrooculography (EOG) signals were measured using the Biosemi Active Two System with 64 EEG-electrodes and 2-EOG electrodes. The EEG electrodes were arranged between the fNIRS sources and the detectors such that the EEG electrodes’ locations were as close to the standard 10–20 system as possible. The EOG electrodes were placed above the right eyebrow (vertical) and beside the left eye (horizontal). The signals were recorded with a computer, which was placed five meters away from the subject. The sampling rate of the system was 1024 Hz. The fNIRS and EEG recording system were synchronized using a CLK supply assembly embedded in the LABNIRS system.

#### fNIRS channels’ and EEG electrodes’ location measurement

Locations of the fNIRS sources, the detectors, and the EEG electrodes were measured using a 3D digitizer system (Fastrak, Shimadzu, Japan). The system had the origin at the center of the chin. Four reference points were obtained at the nasion (Nz), right pre-auricular point (AR), left pre-auricular point (AL), and anterior commissure (Cz). The location of the fNIRS sources, the detectors, and the EEG electrodes were obtained based on the origin and the four reference points. The coordinate of an fNIRS channel was computed from the positions of the sources and detectors using NIRS-SPM software^31^ (Fig. 5b).

### Data preprocessing

**Figure 6:**
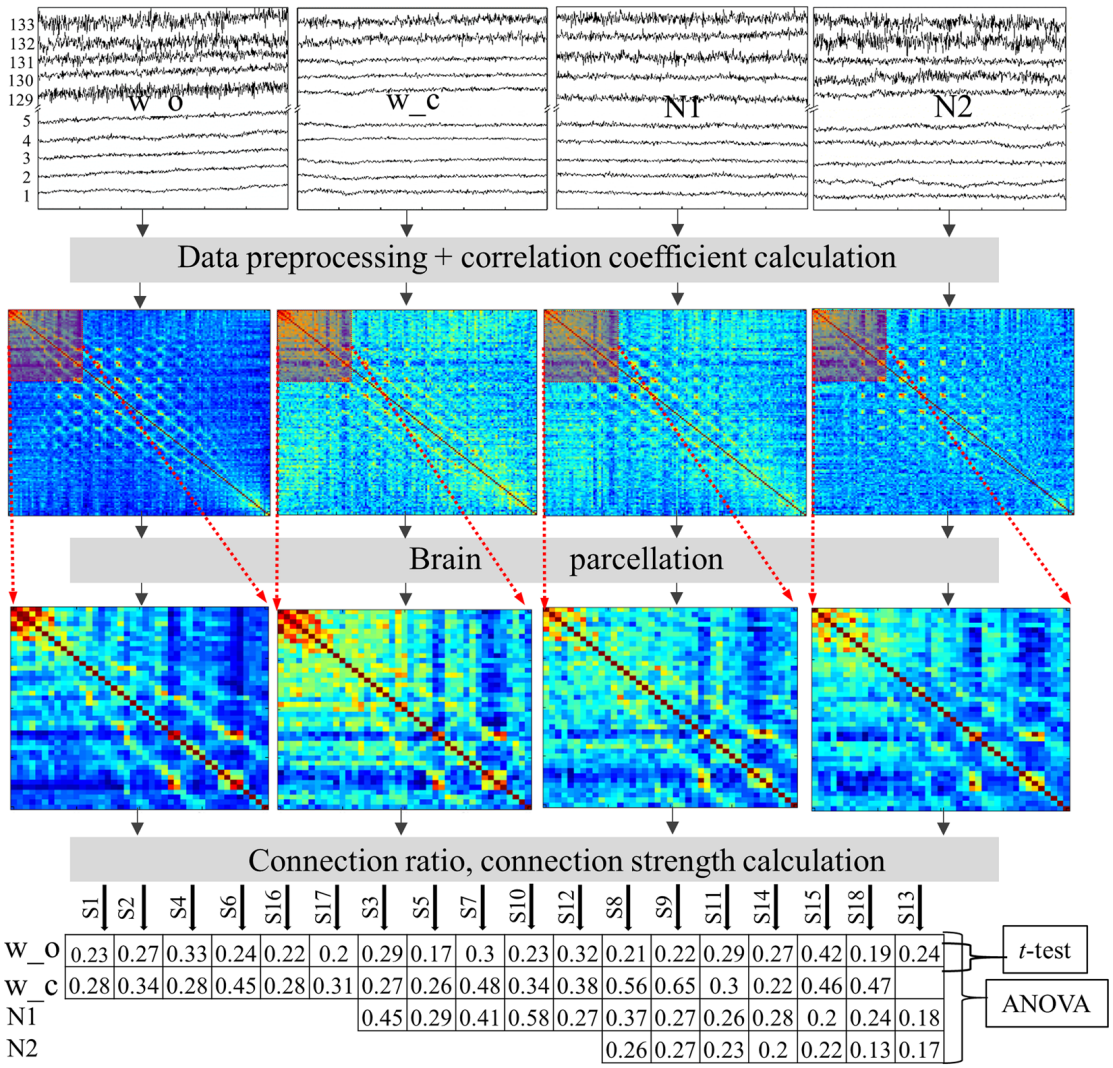
Data processing procedure. Raw data were preprocessed and the Pearson correlation coefficients were calculated, averaged, and normalized to form a symmetric 133 × 133 matrix for a subject in a state with a hemodynamic response. The 133 × 133 matrix was split up into 15 smaller matrixes, corresponding to 15 networks. The connection ratio and connection strength were then calculated for each network. Finally, the ANOVA and post-hoc *t*-test were applied on the connection ratio and connection strength.

The whole data processing procedure is described in Fig. 6. The fNIRS data were preprocessed using Homer2 software^32^. We manually converted a.txt file, the output from the LabNIRS system, to a.nirs file, the input for the Homer2 software. Firstly, a bandpass filter with the frequency range from 0.01 Hz to 0.08 Hz was applied to the raw light intensity. The normal range for the spontaneous hemodynamic change was found to be from 0.01 Hz to 0.1 Hz^1,2^. However, Zaidi *et al*. found a respiration artifact peak at 0.09 Hz^33^; hence, we chose the bandpass range to be 0.01 Hz-0.08 Hz. Secondly, the motion artifact and superficial layer signal were removed using principle component analysis^34^. Thirdly, differential pathlength factor (DPF) values were calculated for three wavelengths (Equation (1)). Finally, the hemodynamic responses including oxy-hemoglobin (HbO), deoxy-hemoglobin (Hb), and total-hemoglobin (THb) were computed from the processed light intensity using the modified Beer-Lamberts’ law.

1 $$DPF\,(\lambda ,\,A)=\alpha +\beta {A}^{\gamma }+\delta {\lambda }^{3}+\varepsilon {\lambda }^{2}+\zeta \lambda $$

where $\alpha =223.3,\,\beta =0.05624$, A: subject mean age (24), γ = 0.8493, δ = −5.723*10^−7^, λ: wavelengths, ζ = −0.9025^35^. Replacing λ by the wavelengths, we got DPF (780 nm) = 6.1, DPF (805 nm) = 5.9, and DPF (830 nm) = 5.5.

The preprocessing algorithm for fNIRS was first performed on the finger tapping task data to examine its efficiency. The unprocessed data from the finger tapping task showed an increase of HbO in many brain regions, while the preprocessed data showed a localized increase of HbO in the left motor cortex during a right finger tapping. After proving its efficiency, the preprocessing algorithm was applied to the rest and sleep data sets. The EEG and EOG signals were preprocessed using EEGLab software^36^. Both the EEG and EOG were bandpass filtered from 0.3 Hz to 35 Hz.

### Sleep stage verification

**Figure 7:**
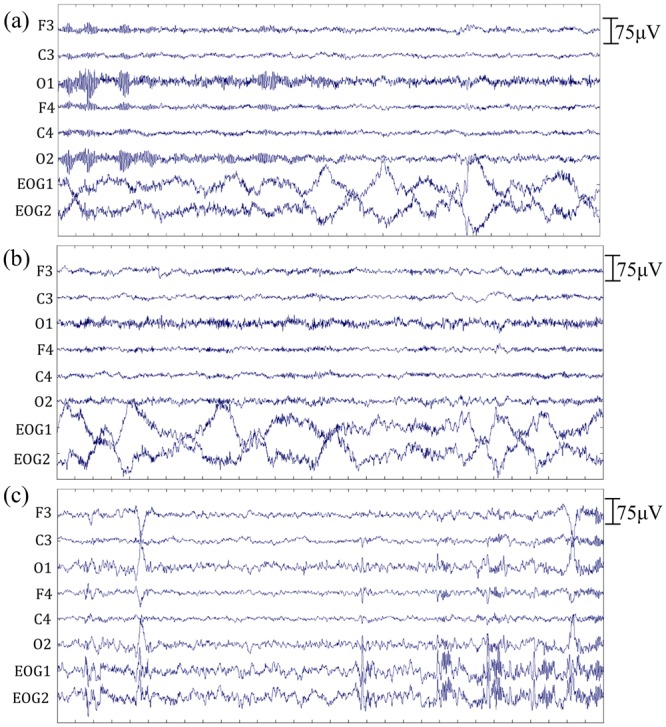
Signals from six EEG electrodes, F3, F4, C3, C4, O1, and O2, and two EOG electrodes (30 seconds) of a subject in three states. (**a**) Quiet wake-eyes closed; (**b**) non-rapid eye movement sleep stage 1; (**c**) non-rapid eye movement sleep stage 2.

Based on the experimental protocol and the sleep scoring results, the subject’s condition was divided into four states: quiet wake-eyes open (w_o), quiet wake-eyes closed (w_c), non-rapid eye movement (NREM) sleep stage 1 (N1), and NREM sleep stage 2 (N2). The w_o state was from the five-minute rest phase, and the other three states were from the ten-minute sleep phase. In order to define the sleep stage, we followed the American Academy Sleep Medicine manual for scoring sleep 2007^37^. The signals from six EEG electrodes, F3, F4, C3, C4, O1, and O2, and two EOG electrodes were used for scoring (Fig. 7). The ten-minute EEG and EOG data were divided into 20 epochs, with each epoch consisting of 30-second data. Every epoch was plotted in a separated graph. Twenty graphs from each subject were arranged, marked, and sent to be scored independently by two experienced medical doctors. The scoring results were similar between two doctors. After a discussion, both doctors rescored the epochs that were scored differently.

### Correlation coefficient calculation

Because the time a subject spent in each state was different (Table 1), fNIRS data was first divided into different consecutive 30-second data epochs. The Pearson correlation coefficients (ρ) between every two fNIRS channels were then calculated for each epoch. The ρ values for a subject, in a state, were the average of all ρ values calculated from the epochs belonging to the state. In order to convert the sampling distribution of the Pearson correlation coefficients to the normal distribution, the ρ values were transformed to the z values using the Fisher z-transformation (Equation (2)).

2 $$z=\frac{1}{2}\,\mathrm{ln}(\frac{1+{\rm{\rho }}}{1-{\rm{\rho }}})$$

### Brain parcellation and network

**Figure 8:**
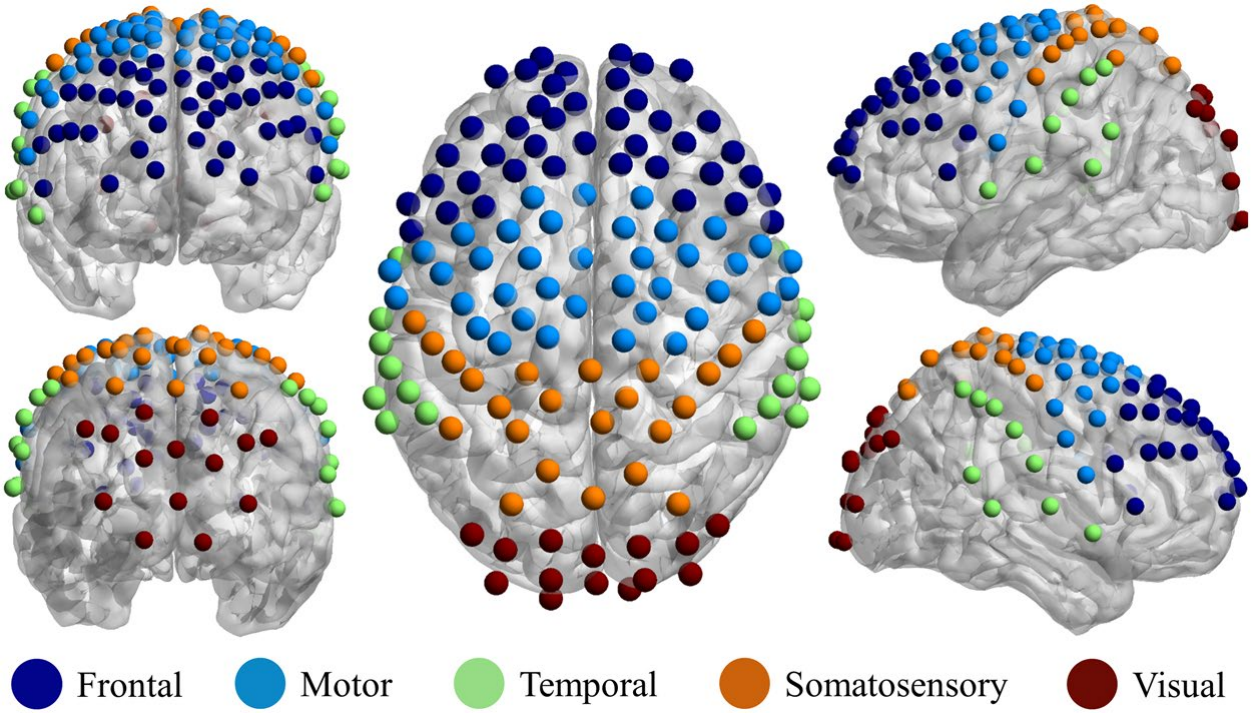
Brain parcellation. The 133 fNIRS channels were grouped into five brain regions; frontal (41 channels), motor (38 channels), temporal (17 channels), somatosensory (23 channels), and visual (14 channels). Brain map images were generated using BrainNet Viewer software^38^.

Based on the fNIRS channels’ locations and Talairach coordinates, each fNIRS channel was first assigned to a Brodmann area using the parcellation function in NIRS-SPM^31^. After that, by utilizing the information of the Brodmann areas, the 133 fNIRS channels were grouped into five brain regions including the frontal (41 channels), motor (38 channels), temporal (17 channels), somatosensory (23 channels), and visual (14 channels) regions (Fig. 8). Finally, 15 cortical networks with 5 within-networks (connection within a brain region such as the frontal network, motor network, etc.) and 10 inter-networks (connection between two brain regions, for example, the frontal-motor network, frontal-temporal network, etc.) were formed from the five regions.

### Connection ratio and connection strength

The number of significant connections of a network was calculated by counting all connections belonging to the network that had an absolute z-value greater than the threshold. In this study, the threshold range was chosen from 0.2 to 0.7. The connection ratio (CR) was then computed as a ratio of the significant connections on the network total connections. The connection strength (CS) of a network was the average of the absolute z-values of all connections.

### Statistical test

The ANOVA tests were first applied to the CR and CS of a network over four states. After that, the post-hoc analyses (*t*-test) were then conducted for the networks and states, which were statistically significant from the ANOVA test. The statistical test was considered statistically highly significant when the p-value was less than 0.001 and statistically significant when the p-value was less than 0.05. Since the statistical test was conducted for 15 networks independently, the Bonferroni correction was applied to counteract the multiple comparison problems. If the corrected p-value was less than 6.7 × 10^−5^, the statistical test was statistically highly significant, and if the corrected p-value was less than 3.3 × 10^−3^, the test was statistically significant.

## Electronic supplementary material

Supplementary information
