# Supplementary material for: Exploring brain functional connectivity in rest and sleep states: a fNIRS study
Source: Sci Rep. 2018 Nov 1;8:16144. doi: 10.1038/s41598-018-33439-2 (PMC6212555; doi:10.1038/s41598-018-33439-2)
Supplement: Supplementary file 1 — Supplementary information [file 41598_2018_33439_MOESM1_ESM.pdf]

Exploring brain functional connectivity in rest and sleep states: an fNIRS study

Thien Nguyen[a], Olajide Babawale[b], Tae Kim[a], Hang Joon Jo[c], Hanli Liu[b*], Jae Gwan Kim[a,d*]

SUPPLEMENTARY FIGURES

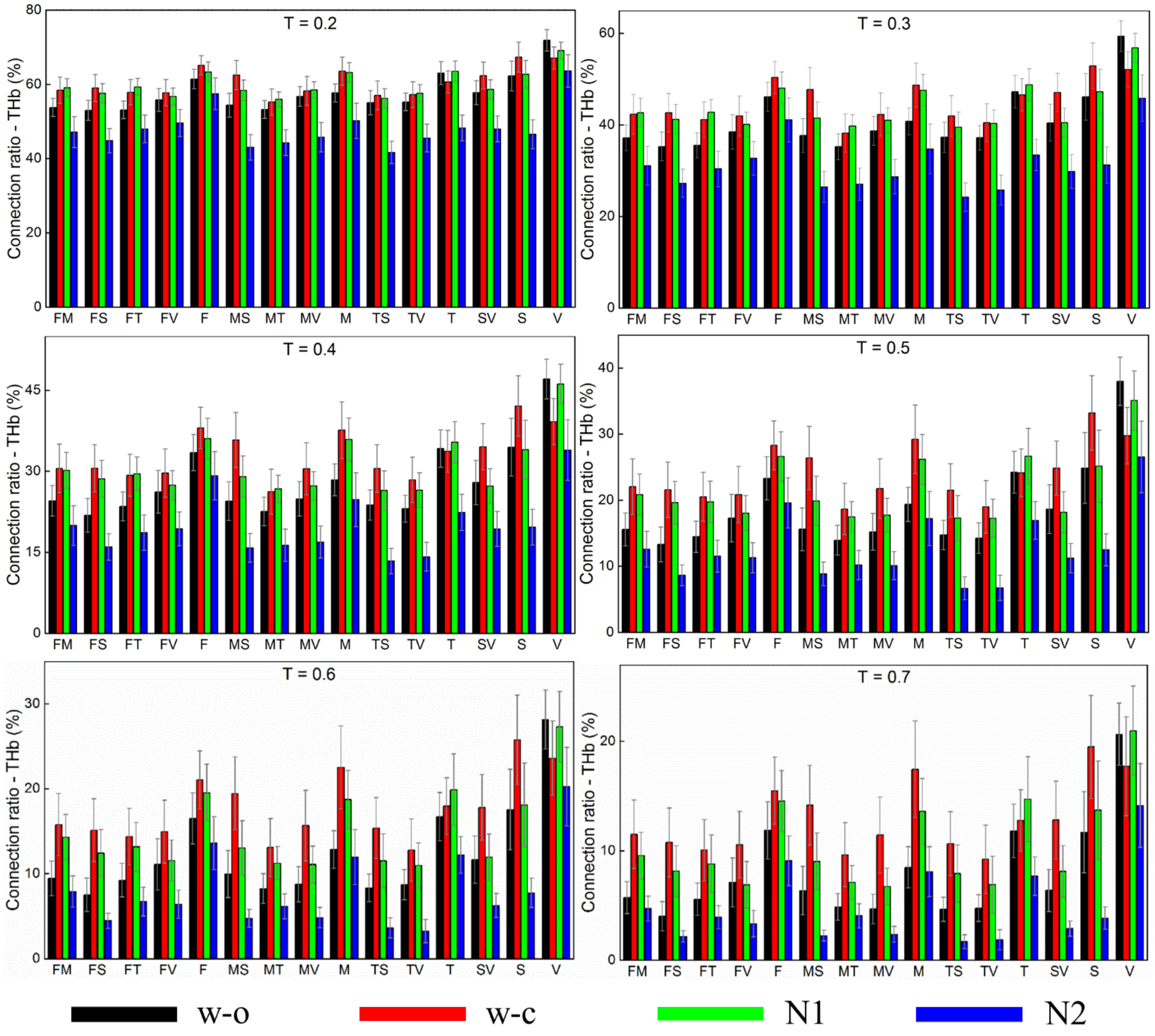

Fig. 1: Connection ratios calculated from THb. The error bar represents the standard error. T (T = 0.2): threshold, F: frontal, M: motor, T: temporal, S: somatosensory, V: visual, FM: frontal-motor.

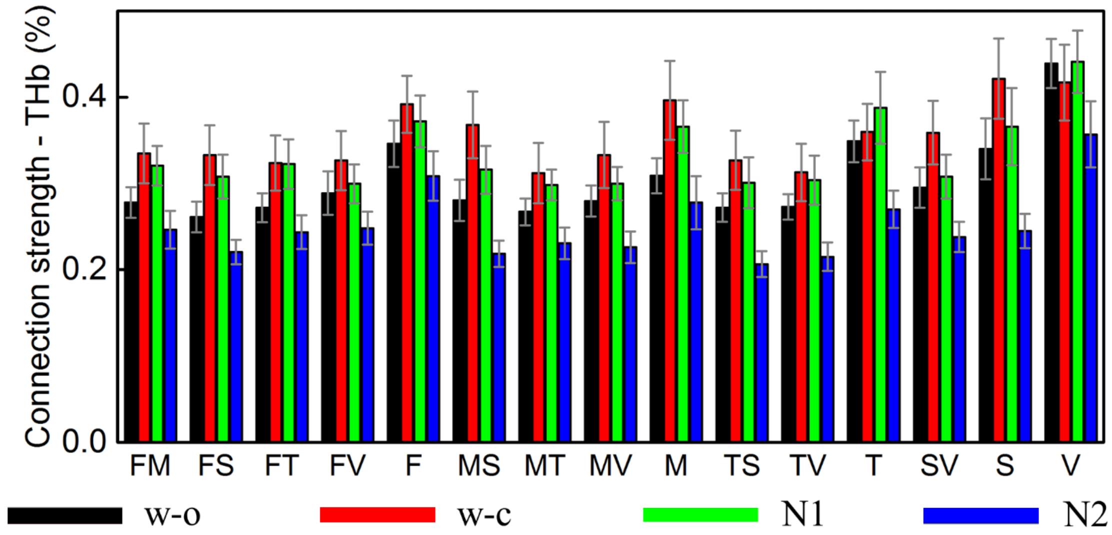

Fig. 2: The averaged connection strength from 18 subjects in 4 states and 15 networks derived from THb. The error bar represents the standard error. F: frontal, M: motor, T: temporal, S: somatosensory, V: visual, FM: frontal-motor.
